# [^223^Ra] RaCl_2_ nanomicelles showed potent effect against osteosarcoma: targeted alpha therapy in the nanotechnology era

**DOI:** 10.1080/10717544.2021.2005719

**Published:** 2022-01-04

**Authors:** Yang Yang, Luciana Magalhães Rebelo Alencar, Martha Sahylí Ortega Pijeira, Beatriz da Silva Batista, Alefe Roger Silva França, Erick Rafael Dias Rates, Ruana Cardoso Lima, Sara Gemini-Piperni, Ralph Santos-Oliveira

**Affiliations:** a Department of Nuclear Medicine, The Second Affiliated Hospital of Zhengzhou University, Zhengzhou, China; b Department of Physics, Laboratory of Biophysics and Nanosystems, Federal University of Maranhão, Maranhão, Brazil; c Brazilian Nuclear Energy Commission, Nuclear Engineering Institute, Laboratory of Nanoradiopharmaceuticals and Synthesis of Novel Radiopharmaceuticals, Rio de Janeiro, Brazil; d Instituto de Ciências Biomédicas, Universidade Federal do Rio de Janeiro, Rio de Janeiro, Brazil; e Zona Oeste State University, Laboratory of Radiopharmacy and Nanoradiopharmaceuticals, Rio de Janeiro, Brazil

**Keywords:** Nano-radiotherapy, nano-radium, radium dichloride ([^223^Ra] RaCl_2_) Nanoemulsion, nanomicelles, cancer, bone

## Abstract

The treatment of bone metastatsis as primary bone cancer itself is still a challenge. The use od radium dichloride ([^223^Ra] RaCl_2_) has emerged in the last few years as one of the best treatment choice for bone cancer, with especial focus in bone metastasis. The alpha-emitter radiopharmaceutical has showed potent and efficient results in several clinical trials. In this study we have formulated radium dichloride ([^223^Ra] RaCl_2_) nanomicelles in order to evaluate and compare with pure radium dichloride ([^223^Ra] RaCl_2_). The results showed that nanomicelles at the same dose had a superior effect (20% higher efficient) when compared with pure radium dichloride ([^223^Ra] RaCl_2_). The results corroborated the effectiveness of the nanosystem validating the application of nanotechnology in alpha-radiotherapy with radium dichloride ([^223^Ra] RaCl_2_).

## Graphical Abstract

**Figure:**
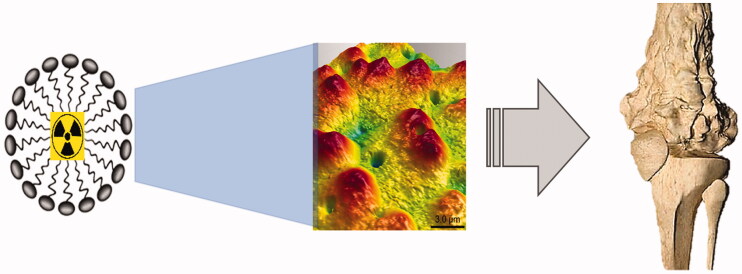

## 1. Introduction

The radium dichloride ([^223^Ra] RaCl_2_) is an alpha emitter radiopharmaceutical used in targeted alpha therapy (TAT) to selectively bind to bone cancer/metastases sites (Hosono et al., 2019) killing selectively, the tumor cells by inducing DNA breaks in a potent and localized manner (Morris et al., 2019). The radium [^223^Ra] is bone-seeking calcium mimetic, which bonds into the newly formed bone stroma, especially osteoblastic or sclerotic metastases. The ability to emit high-energy alpha particles of short-range (<100 μm/10 cell diameters) (Parker et al., 2013; Du et al., 2017; Corrêa et al., 2021) endows short penetration, promoting localized cytotoxic effect with shallow toxic effects on adjacent healthy tissue (Liepe, 2009; Suominen et al., 2019). This feature allows the bone cancer/metastases treatment, sparing the bone marrow region.

Nanomicelles are colloidal nanosystems formed of amphiphilic monomers composed of two parts, a small hydrophobic head and a long hydrophilic tail (Trinh et al., 2017). The nanomicelles, are widely used to improve: (i) blood circulation time (Abbina et al., 2020), (ii) structural stability (Ree et al., 2017), (iii) controllable size (Tawfik et al., 2020), (iv) bioavailability (Joseph et al., 2017), (v) sensitivity (especially for imaging agents) (Cheng et al., 2016) and (vi) specificity (of various therapeutic agents) (Pawar et al., 2018).

In the case of therapeutics agents, properly loaded into nanomicelles, due to controllable size property and using, in the case of tumors, the EPR (Enhanced Permeability and Retention) effect is possible to achieve selective and increased accumulation in tumor site (Sun et al., 2018). Thus, the damage to other organs is remarkably reduced. Finally, therapeutics agents incorporated into nanomicelles can also be protected against biodegradation as providing a sustained drug release, leading to increased drug efficacy (Nogueira et al., 2016; Ferro et al., 2017; Dos Santos Matos et al., 2020; Schuenck-Rodrigues et al., 2020; Magne et al., 2021).

Another important aspect related to nanomicelles is the excretion route. In most cases, nanomicelles are excreted in the urine by the renal via (Wang et al., 2018; Helal et al., 2021). The renal via is the faster and most secure via. Using the renal system to excrete the radium dichloride ([^223^Ra] RaCl_2_) represents a significant achievement and may increase the safety aspects related to the use of this radiopharmaceutical routinely.

In this study, we have produced and characterized radium dichloride ([^223^Ra] RaCl_2_) nanomicelles as evaluated in vitro to compare with the radium dichloride ([^223^Ra] RaCl_2_) solution. We demonstrated that 127-Pluronic-[^223^Ra] RaCl_2_ nanomicelles showed a dose-response behavior and an increased effect on osteosarcoma cells, decreasing the cell viability more efficiently.

## 2. Materials and methods

### 2.1. Reagents

Phosphate buffered saline (PBS), PBS/EDTA, bovine serum albumin (BSA), methylated bovine serum albumin (mBSA), Freund’s complete adjuvant, Histopaque reagent, Pluronic F127, TRAcP staining kit, DMEM high glucose, Bovine fetal serum, M-CSF, RANK-L, Doxorubicin, Poly-D-lysine, Glucose, HEPES, Calcium, and Magnesium were purchased from Sigma Aldrich (St. Louis, MO, USA). MayGrünwald and Giemsa dyes were purchased from Merck (Germany). 3% sodium pentabarbital (Hypnol^®^) was purchased from Syntec (Brazil). Tevametho (injectable metrotrexate 25 mg/mL) was purchased from Teva Farmacêutica Ltda (Brazil), and Hydroxychloroquine was purchased from Must Check. The radium dichloride (Xofigo^®^) was purchased from Bayer.

### 2.2. Preparation of the nanomicelles

A mass of 1 mg/mL of [223Ra] RaCl2 which corresponded to a total dose of 2µCi, was weighed and added to the micellar dispersion of Pluronic F127. The system was gently stirred using a magnetic bar (Magnetic Stirrer, IKA, C-MAG HS-7) for 5 min and then processed for 5 min using an ultrasonic processor (UP100H, Hielscher, Power: 100%, Cycle: 1) under an ice bath at 10 °C. The dispersion of polymeric nanomicelles containing [^223^Ra] RaCl_2_was stored in an amber flask for further analysis and refrigeration (2–8 °C).

### 2.3. Particle size

The particle size, size distribution, and polydispersity index (PDI) of the nanosystem were determined by dynamic light scattering (DLS) using the equipment Zetasizer Nano ZS (Malvern Instruments, UK). Measurements were performed in triplicate at 25 °C, and the laser incidence angle in relation to the sample was 173° using a 12 mm^2^ quartz cuvette. The mean ± standard deviation (SD) was assessed.

### 2.4. Atomic force microscopy (AFM)

The AFM analysis has been performed using a Multimode 8 microscope (Bruker, Santa Barbara, CA). Two central studies have been conducted in the sample:127-Pluronic-[^223^Ra] RaCl_2_ nanomicelles. The morphology and topography of the nanomicelles were analyzed. For these measurements, Scanasyst Air probes were used, with a nominal tip ratio of 2 nm and nominal spring constant of 0.4 N/m. However, the actual spring constant was calibrated by the thermal noise method. A drop of the nanomicelles solution was deposited in freshly cleaved mica to form the nanomicelles film. The scanning mode used was Peak Force Tapping Quantitative Nanomechanics (QNM), with a resolution of 256 × 256 lines per scan and scan frequency of 0.5 Hz.

### 2.5. *In vitro* cytotoxicity

#### 2.5.1. Cell culture

SAOS2 cells were plated in a density of 1 × 10^4^ cells/well for 24 h. The cells were maintained in DMEM/D-glucose (high glucose) medium, supplemented with 10% FBS, penicillin (0.5 U/mL) and streptomycin (0.5 mg/mL). Cells were incubated at 37 °C in a humidified atmosphere of 5% CO2. Cells were grown to confluence in 75 cm^2^ culture flasks and were detached by brief treatment with trypsin (0.1%)/EDTA (0.01%).

### 2.6. MTT – viability assay

**Figure 1.**
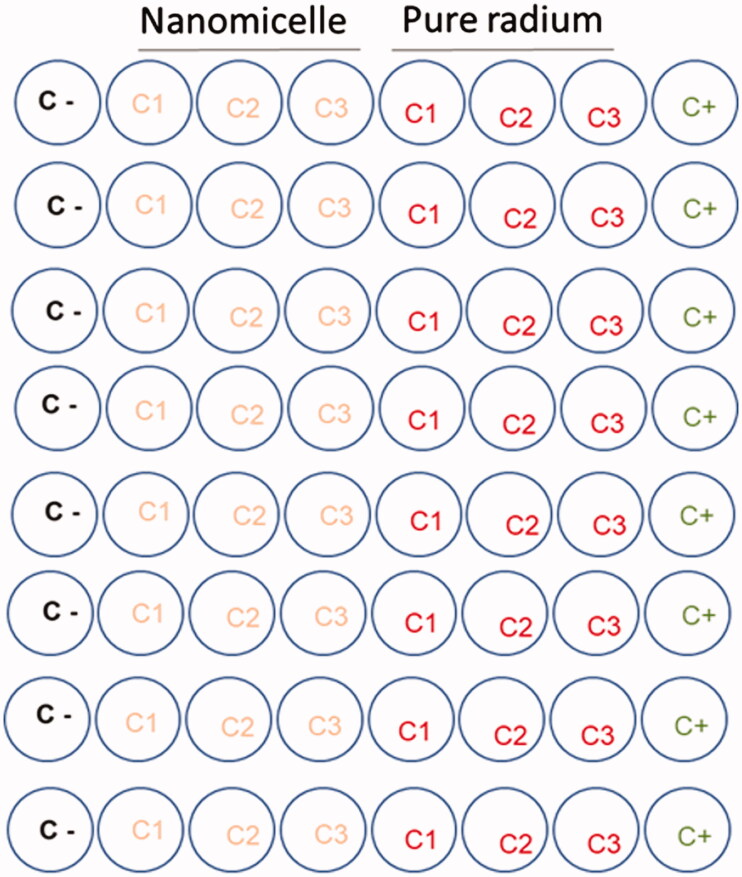
Schematic assay of MTT test in SAOS2 cells using nanomicelles (127-Pluronic-[^223^Ra] RaCl_2_]) compared to pure radium dichloride ([^223^Ra] RaCl_2_]), the negative (C−) and the positive (C+) control.

SAOS2 (1 × 10^4^ cells/well) were seeded and allowed to attach for 24 h. The cells were divided into two groups: control group (pure [^223^Ra] RaCl_2_) and intervention group (127-Pluronic-[^223^Ra] RaCl_2_] nanomicelles) in three distinct activities: C1: 1 µC, C2: 0.5 µC, and C3: 0.12µCi) as demonstrated in Figure 1. As a positive control (cell death, C+) cell was exposed to 1% sodium dodecyl phosphate to lyse cell membrane.

After 24 h, MTT assay was realized to determine cell viability. The redox mitochondrial potential in metabolically active live cells reduce MTT to a formazan crystal. The strongly purple pigmentation of formazan product is dissolved in organic solvent DMSO and measured at 570 nm to determine live cells. The more purple, the more living cells are present. All data were expressed as a fold increase of negative control (cells without treatment) as the mean ± standard deviation of three independent experiments with *p < 0.05 and **p < 0.005 indicates a significant difference *vs* negative control.

## 3. Results

### 3.1. Particle size

**Figure 2.**
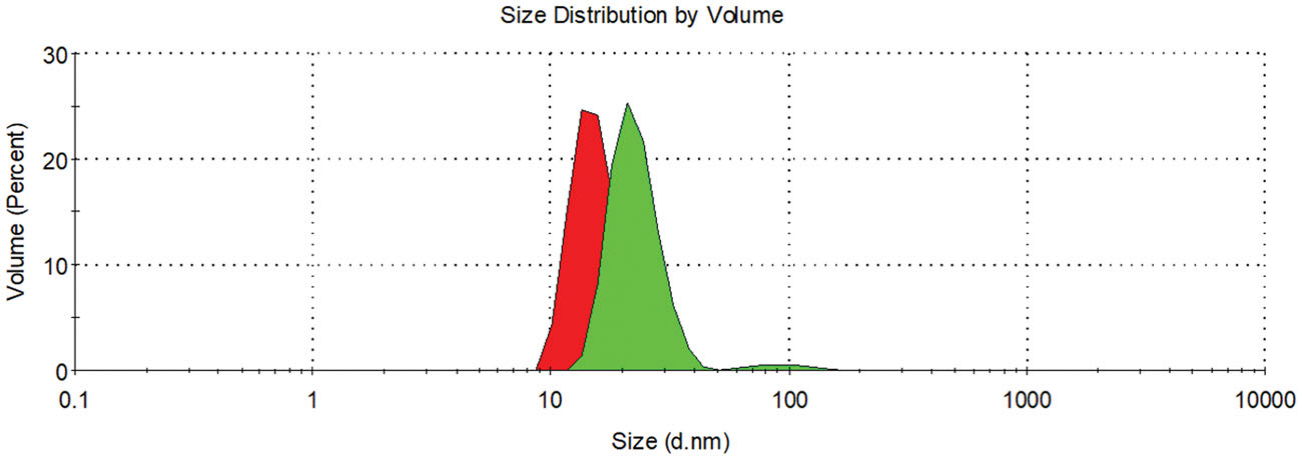
Two independent assays of DLS. First (in red) freshly produced nanomicelles. Second (in green) 1-week stored nanomicelles.

The DLS assay demonstrated that the freshly produced 127-Pluronic-[^223^Ra] RaCl_2_] nanomicelles showed a mean size of 129.4 nm with a PDI of 0.303. After one week stored in the refrigerator, the 127-Pluronic-[^223^Ra] RaCl_2_] nanomicelles showed a mean size of 169.4 and a PDI of 0.381 (Figure 2).

### 3.2. Ultrastructural characterization

**Figure 3.**
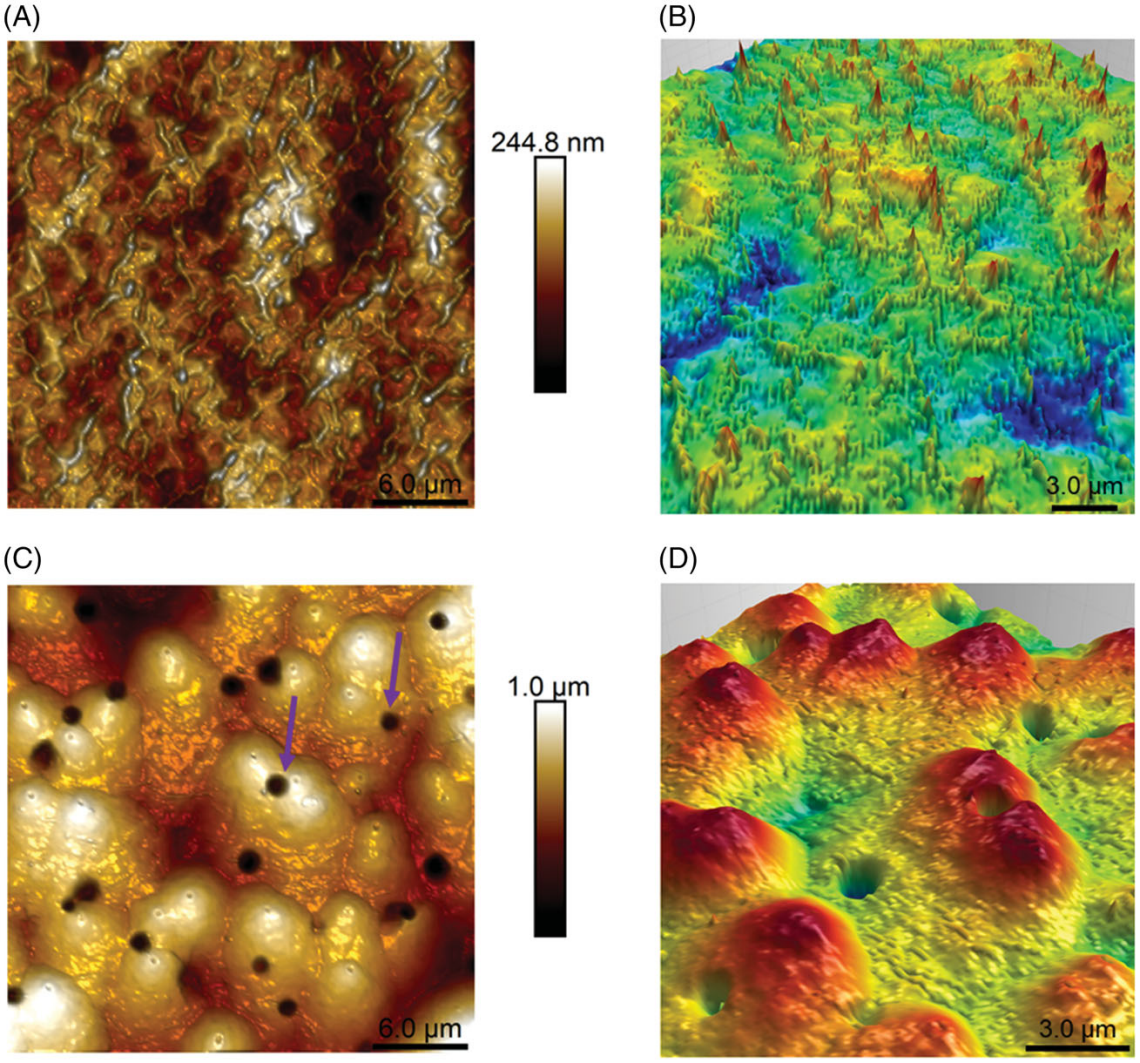
Atomic force microscopy height map of the white (127-Pluronic) nanomicelles (A) and 127-Pluronic-[223Ra] RaCl_2_ nanomicelles (C) and their respective three-dimensional topographies (images B and D) acquired in the central region of heigh maps. The purple arrows (C) highlight dips in the film surface possibly caused by alpha particle emission.

The ultrastructure of the 127-Pluronic-[223Ra] RaCl_2_ micellar film was investigated by AFM and compared with the 127-Pluronic empty nanomicelles sample (Figure 3). Figure 3(A) shows a 127-Pluronic white nanomicelles film. Polymeric chain structures with a diameter of 263.4 ± 12.1 nm are observed. The maximum film height is 244.8 nm. The three-dimensional representation of the central region of Figure 3(A) is shown in Figure 3(B), in which the polymer chain structures are evident.

Figure 3(C) shows the AFM height image of 127-Pluronic-[223Ra] RaCl_2_ film. It is possible to observe spherical structures whose heights reach 1 μm, evidencing the [223Ra] RaCl_2_. Such structures suggest the filling of 127-Pluronic nanomicelles with [223Ra] RaCl_2_. It is also possible to observe invaginations on the film surface (purple arrows). These depressions do not disrupt the globular structure (Figure 3(D)), keeping the bulkier regions of the film stable, where there is probably a concentration of 127-Pluronic- [223Ra] nanomicelles. Since these depressions are only observed in 127-Pluronic-[223Ra] films, they can be promoted by emitting alpha particles from the 127-Pluronic-[223Ra] RaCl_2_ nanomicelles. However, this emission is not able to destabilize the nanomicelles clusters of 127-Pluronic-[223Ra].

### 3.3. Viability assay

**Figure 4.**
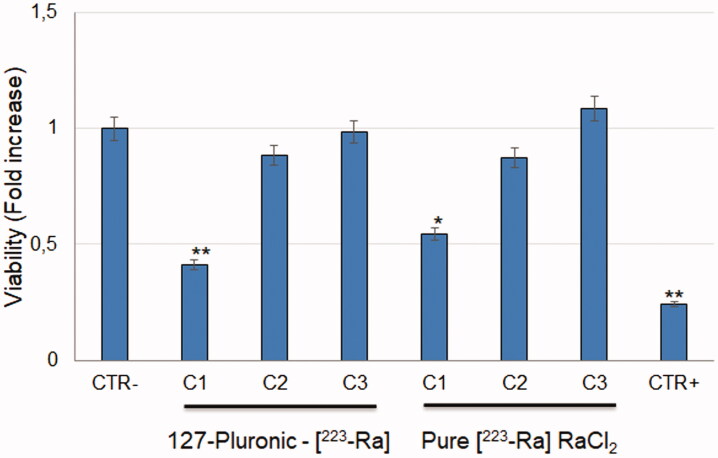
MTT assay of SAOS2 cells exposed to 127-Pluronic-[^223^Ra] RaCl_2_] nanomicelles and pure[^223^Ra] RaCl_2_ using three different activities (C1: 1 µC, C2: 0.5 µC, and C3: 0.12µCi). The positive control (cell death, C+) was perfomed using 1% sodium dodecyl phosphate to lyse cell membrane. Data is expressed as mean ± standard deviation of three independent experiments with *p < 0.05 and **p < 0.005 indicates a significant difference *vs* negative control.

The viability assay (Figure 3) with 127-Pluronic-[^223^Ra] RaCl_2_] nanomicelles showed a dose-dependent response. The same has been observed using pure [^223^Ra] RaCl_2_. The 127-Pluronic-[^223^Ra] RaCl_2_] nanomicelles are more efficient in killing SAOS2 cells when compared with pure [^223^Ra] RaCl_2_ (Figure 4).

## 4. Discussion

The size of 129.4 nm is the expected range for the treatment of bone cancer or bone metastases. For instance, Reissig et al. (Reissig et al., 2019) developed barium sulfate nanoparticles doped with [^224^Ra]Ra(NO_3_)_2_ as a palliative treatment for bone pain with a size of 140 nm.

It is essential to notice that the storage of the 127-Pluronic-[^223^Ra] RaCl_2_ nanomicelles just slightly changed the size and the PDI value. This happened probably due to a deficient aggregation process caused by an intermicellar aggregation (Ree et al., 2017). A vital factor must be considered in this intercurrence: the continuous emission of alpha particles. With an energy of 5 MeV, the [^223^Ra] will cause a large amount of ionization (Guinn, 2003), which may cause a destabilizing event, inducing the intermicellar collapse and further aggregation.

In this study, we have assumed the entrapment efficacy of 100% encapsulation since we have used the nanomicelles bulk without any further treatment (Jaiswal et al., 2015; Raval et al., 2017; Carvalho et al., 2019; Meng et al., 2020).

The PDI value showed a monodispersive behavior of the nanosystem. According to Danaei et al. (2018), a polydispersity index (PDI) value varying from 0.1 to 0.5 for pharmaceutical products may be considered a monodisperse system. Controversially, Han and Jiang (Jiang et al., 2020) stated that a PDI value higher than 0.1 but lower than 0.3 could be defined as monodispersive. Thus, considering that the 127-Pluronic-[^223^Ra] RaCl_2_] nanomicelles showed a PDI of 0.303, we assumed that a medium dispersity behavior is the most appropriate denomination (Wigner et al., 2021).

The use of [^223^Ra] RaCl_2_ has some limitations (Sartor and Sharma, 2018). The main downside is that [^223^Ra] RaCl_2_ only targets the bone, disabling your application for other types of cancer. According to Morris et al. (2019), the conjugation of alpha particle-emitting radionuclides with tumor-targeted carrier molecules, including antibodies, peptides, and small molecules, has the potential to deliver tumor-specific targeting and overcome the main limitation of [^223^Ra] RaCl_2_. In this scenario, the development of 127-Pluronic-[^223^Ra] RaCl_2_] nanomicelles may represent the most appropriate strategy. For instance, Huang et al. (2019)have developed dual-targeting nanomicelles with CD133 and CD44 aptamers for lung cancer. The results demonstrated that the nanosystem developed was able to increase the therapeutic effect against lung cancer. In this sense, Xie et al. (2020) showed that a nanomicelles nanosystem composed of 3D6 antibody fragments and decorated with glucose was capable of delivering 3D6 antibody fragments, inhibiting Aβ aggregation in the brain tumor cell, by using the recycling glucose transporter (Glut)-1 proteins.

Thus, considering the studies mentioned above is possible to think about the design of 127-Pluronic-[^223^Ra] RaCl_2_ nanomicelles decorated or co-loaded with other compounds creating a unique dual-mode nanosystem for cancer therapy.

It is well known that [^223^Ra] RaCl_2_ is unlikely to develop resistance mechanisms observed with other anticancer therapies since the main target is the DNA itself (Morris et al., 2019). According to Wei et al. (2015), nanomicelles can effectively enhance drug potency and combat drug resistance by promoting cellular uptake and decreasing efflux of the anticancer drug, creating a very potent anti-drug resistance drug.

Finally, the 127-Pluronic-[^223^Ra] RaCl_2_ nanomicelles showed a superior effect to pure [^223^Ra] RaCl_2_ in bone cancer cells. This may be explained by the fact that nanomicelles have facilitated intracellular trafficking (Lu et al., 2015; Li et al., 2020; Yu et al., 2021), reaching faster the cytoplasm and the nucleus of the cells and causing more efficient DNA double-strand breaks.

## 5. Conclusion

The use of 127-Pluronic-[^223^Ra] RaCl_2_ nanomicelles may represent a significant advance in the field of radiopharmacy and nuclear medicine. Our data demonstrated that it is possible to create stable nanosystems (127-Pluronic-[^223^Ra] RaCl_2_nanomicelles), which has a superior effect against bone cancer cells when compared with pure [^223^Ra] RaCl_2_. Also, the 127-Pluronic-[^223^Ra] RaCl_2_ nanomicelles may be decorated and incorporated with a great variety of agents and compounds (monoclonal antibodies, aptamers, peptides…), overcoming the limited use of [^223^Ra] RaCl_2_.
